# Green Extraction and NMR Analysis of Bioactives from Orange Juice Waste

**DOI:** 10.3390/foods14040642

**Published:** 2025-02-14

**Authors:** Paula Scarabotto Penteado, Maria Carolina B. Di-Medeiros Leal, Maria Gabriela Aparecida Carosio, Alef dos Santos, Mateus Lodi Segatto, Daniel Petinatti Pavarini, Danielle Fernandes da Silva, Jéssica Cristina Amaral, Maria Fátima das G. F. da Silva, Vânia G. Zuin Zeidler, Antonio G. Ferreira

**Affiliations:** 1 Department of Chemistry, Federal University of São Carlos, São Carlos 13565-905, SP, Brazilgiba@ufscar.br (A.G.F.); 2 Department of Chemistry, University of Wisconsin-Madison, Madison, WI 53706, USA; m.gabbiiii@gmail.com; 3 Perdue Animal Nutrition, Salisbury, MD 21804, USA; 4 Institute of Sustainable Chemistry, Leuphana University Lüneburg, 21335 Lüneburg, Germany

**Keywords:** microwave-assisted extraction, GC–MS, flavonoids, circular economy, environmental health

## Abstract

Brazil is a global leader in the orange industry, producing approximately one-fourth of the world’s oranges and generating over 50% of the associated waste. These by-products are rich in bioactive compounds; however, their improper disposal poses environmental risks. This study employs an eco-friendly approach—microwave-assisted extraction—to recover valuable compounds from orange juice production waste. The extracted compounds were analyzed using nuclear magnetic resonance (NMR) and gas chromatography–mass spectrometry (GC–MS). Key bioactives, including D-limonene, valencene, hesperidin, and carbohydrates, were successfully identified. NMR effectively traces and semi-quantifies these compounds, while microwave-assisted extraction enables the sustainable recovery of high-purity hesperidin, confirmed by NMR (87.66%) and HPLC (84.30%) analyses.

## 1. Introduction

According to the latest global citrus market analysis by the United States Department of Agriculture (USDA), Brazil is the world’s leading producer of both oranges and orange juice, followed by China and Mexico [1]. The juice production sector plays a crucial role in the food industry, ensuring year-round availability despite the seasonal nature of fruit production. Among fruit juices, orange juice remains the most widely consumed. The citrus juice industry, particularly involving *Citrus sinensis* (‘Pera’ sweet orange) grafted onto *Citrus limonia* (‘Rangpur’ lime) [2], produces not only juice and essential oils as primary products but also generates significant amounts of waste. This waste, with its economic potential, offers opportunities to cut costs and reduce environmental impact. This is particularly true when valuable by-products are identified and extracted using eco-friendly processes that minimize environmental impact [3]. Approximately 50% of the total production mass is classified as waste, yet it is rich in various high-value compounds [4]. These include soluble carbohydrates (cellulosic, pectic, and starch polysaccharides) from the flavedo, albedo, and seeds; terpenes (such as D-limonene, myrcene, valencene, and linalool) from the peels; and flavonoids, including hesperidin [5]. The growing interest in valorizing these compounds has driven industrial research toward sustainable extraction methods [6].

Orange juice is the primary product of the citrus industry, but its processing generates substantial liquid and solid waste, contributing to significant biomass. Many industrial operations aim to mitigate environmental challenges while extracting economic value from these by-products through re-extraction, separation, and purification processes. Essential oils, such as D-limonene and valencene, are valuable derivatives of orange processing. However, maintaining their quality requires the removal of impurities like phospholipids, pigments, and fatty acids [7]. Despite the large volume of waste produced during citrus processing, these by-products contain bioactive molecules with antimicrobial, anti-allergic, anti-diabetic, and anti-cancer properties, making them highly valuable for nutraceutical applications in health and nutrition [8]. Beyond nutraceuticals, citrus waste is also utilized in pharmaceuticals, food production, fertilizers, and agricultural pesticides.

**Figure 1:**
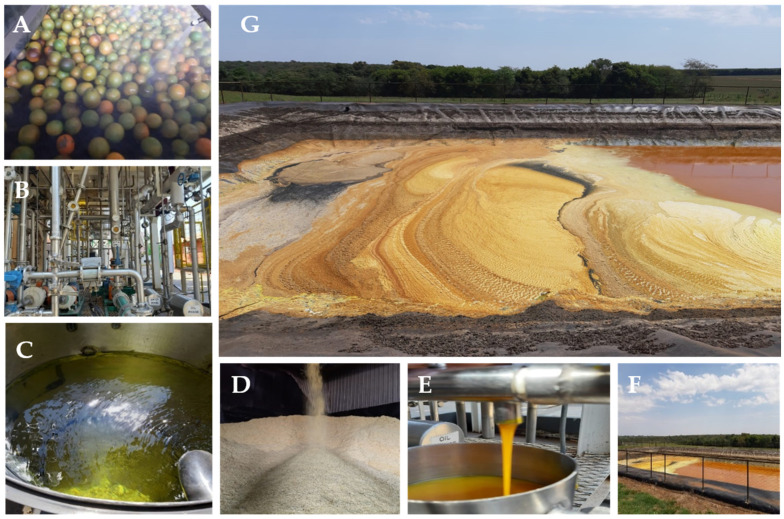
Images of the industrial production stages of orange juice and its wastes. (**A**) Selection and washing of the orange fruits. (**B**) Separation ducts of the oily and aqueous phases. (**C**) D-limonene tank. (**D**) Orange pomace bran. (**E**) Rich oil mixture tank. (**F**) “Yellow water” waste treatment pond. (**G**) Colloidal suspension present in the “yellow water” waste treatment lagoon. Photos courtesy of the company Agroterenas, Santa Cruz do Rio Pardo, SP, Brazil.

The industrial processing of residual orange biomass is illustrated in Figure 1. The process begins with biomass concentration, followed by ultracentrifugation within the polishing system to obtain a mixture enriched with reusable compounds. Next, the biomass is separated into aqueous and solid waste phases. The aqueous waste, known as “yellow water”, is sent to a fermentation pond, while the solid waste is dried and ground for use as animal feed. During the polisher concentration stage, a de-oiler extracts essential oils from the biomass. These oils then undergo fractionation, purification, and storage. The remaining biomass is further processed, with wastewater sent to treatment ponds and solid particulate matter converted into bran [9].

Hesperidin, a valuable by-product of citrus waste, has been widely studied for its antioxidant, anti-inflammatory, anti-cancer, and anti-aging properties [10]. However, it poses operational challenges in the juice industry due to its undesirable sensory attributes and its tendency to cause equipment blockages. Despite these issues, hesperidin remains economically significant, with its market value varying based on purity [11]. This has driven extensive research and development in the field. The industrial recovery of valuable compounds from citrus waste involves multiple stages, each yielding varying concentrations of these compounds. Optimizing extraction, purification, and characterization techniques is crucial for enhancing efficiency and supporting sustainability and circular economy initiatives. Accurate identification and quantification of target compounds, such as hesperidin, are particularly important, especially when minimal chemical derivatization is required [12]. Citrus processing waste, including peels, seeds, and pulp, is typically sent to collection and treatment facilities, with “yellow water” being the primary residual by-product.

Various strategies have been proposed for recovering bioactive compounds from citrus waste, including phenolic compounds, pigments, oils, lipids, pectin, hesperidin, and cellulose. These strategies involve both direct recovery processes and the use of waste in fermentation to produce biomethane and alcohol, offering environmentally sustainable solutions [4]. For instance, biofuels such as bioethanol and biogas can be produced through ethanolic fermentation, followed by the anaerobic digestion of orange peel waste, leveraging its high sugar and polysaccharide content [13]. Additionally, citrus waste is widely utilized in the production of citrus-derived cellulose [14] and serves as a significant source of essential oils [15]. Natural pigment extracts from sources such as animals, minerals, microbes, and plants are gaining increasing attention from both industry and researchers for their potential as safer colorants in the food industry [16]. These pigments can be recovered from various fruits, including berries, melons, and citrus pomace [16,17].

Traditional extraction techniques, such as liquid–liquid extraction (LLE), ultrasound-assisted extraction (UAE), and supercritical fluid extraction (SFE), are valued for their simplicity and low equipment costs. However, these methods often have significant limitations, particularly long extraction times [18]. In contrast, microwave-assisted extraction (MAE) offers several advantages, including rapid heating, shorter processing times, reduced solvent consumption, improved extraction efficiency, and higher yields [19]. Additionally, microwaves (MW) penetrate the plant matrix, generating heat directly within the cells, which promotes cell disruption and enhances the dissolution of target compounds into the extraction solvent [20].

The samples collected throughout the industrial process contain complex mixtures of compounds, making analysis by GC–MS and HPLC-DAD difficult without prior extraction and purification. In this context, nuclear magnetic resonance (NMR) is the preferred analytical tool because it requires minimal sample preparation, is non-destructive, and effectively characterizes compounds within mixtures [21].

This study aimed to analyze both the quantity and composition of compounds in the waste generated during orange juice production, with a particular focus on hesperidin. To achieve this, various analytical techniques were employed, including NMR, high-performance liquid chromatography (HPLC), and gas chromatography–mass spectrometry (GC–MS). These methods enabled the tracking of compounds throughout the production process and helped identify areas with high concentrations for further investigation. These methods enabled the tracking of compounds throughout the production process and helped identify areas with high concentrations for further investigation. Additionally, microwave-assisted extraction was employed to enhance the sustainability of the process since MAE is an eco-friendly extraction method. By emphasizing the development of green analytical methods for valorizing food waste, this research contributes to scientific knowledge and advances sustainable food technology.

## 2. Material and Methods

### 2.1. Citrus Waste Samples

The sampling process was conducted in September 2019 at the industrial facility located on the Agroterenas farm in Santa Cruz do Rio Pardo, São Paulo, Brazil. Samples were collected from nine stages of the industrial orange juice production process, excluding the final orange juice product. Specifically, samples were obtained from the oil extraction plant—polisher concentration stage. Additionally, three types of bagasse samples, differing in granulometry and the distribution of anatomical fruit parts (peel, albedo, and seed waste), were collected from the residual orange biomass. Aqueous fractions were also collected from the effluent management plant. Figure S1 in the Supplementary Material illustrates the stages of residual biomass treatment and sample collection.

### 2.2. Preparation of Extracts from Waste Samples for NMR Analysis

The chemical constituents in the orange bagasse extraction process and effluent management samples were subjected to liquid–liquid extraction using a 1:1 *v*/*v* mixture of CDCl_3_ and H_2_O (1 mL). The resulting phases were separated and analyzed sequentially by NMR. For the aqueous fraction, 0.1 mL of D_2_O containing a stock solution of TMSP-d_4_ (0.2% *w*/*w*) was added, and the sample was also analyzed by NMR. Additionally, all aqueous fraction samples were lyophilized and subsequently solubilized in 0.5 mL of DMSO-d_6_ for further NMR analysis. A flow chart illustrating this process is provided in Figure S1 of the Supplementary Material.

### 2.3. Preparation of Citrus Essential Oil for NMR Analysis

For NMR analysis, 0.3 mL of orange essential oil sample from the polisher concentration stage of the industrial process was dissolved in 0.1 mL of deuterated chloroform (CDCl_3_ containing TMS), and the resulting solution was transferred to NMR tubes.

### 2.4. Bagasse Analysis for Hesperidin Extraction and Statistical Planning

Three types of bagasse were collected, each differing in particle size and the distribution of fruit components (peel, albedo, and seed residues) as processed by the industry. These were classified as bagasse with liquor (BL), pomace (BC), and peel (CA). To optimize the microwave-assisted extraction (MAE) of hesperidin from orange bagasse and determine which waste source had the highest flavonoid concentration, an experimental design was implemented. The procedure, guided by previous research [3], aimed to maximize extraction efficiency by varying key parameters. The experimental design was structured to determine the optimal extraction efficiency by quantifying the concentration of hesperidin. The isothermal extraction time ranged from 10 to 40 min, with intervals at 10, 25, and 40 min, while temperatures were tested at 40 °C, 60 °C, and 80 °C, as shown in Table 1 [22]. Note that this solvent composition is unfavorable for sugar extraction.

After extraction, the samples were analyzed using a UHPLC Waters (Milford, MA, USA) H-Class system equipped with a C8 Acquity BEH column (1.7 µm, 2.1 × 50 mm). Detection was performed with a photodiode array detector (PDA Waters (Milford, MA, USA) H-Class) set at 280 nm. The mobile phases consisted of A (ultrapure water) and B (acetonitrile), with a flow rate of 0.3 mL/min.

The parameters used for the calibration curve with the hesperidin standard were as follows: stock solution: 400 mg/L; 7 concentration points: 400, 300, 200, 100, 60, 20, and 10 mg/L; injection curve: y = 4420.13x − 20,815.08 (R^2^ = 0.9999); LOD = 4.16 mg/L; LOQ = 13.88 mg/L. The calibration curve is presented in Figure S5 of the Supplementary Material.

The chromatographic quantification data (Table 1) were used for statistical analysis (Table S1) and generated the response surface, which highlighted the optimal flavonoid extraction conditions and identified the best matrix source.

The statistical analysis, illustrated through Pareto charts and response surfaces (Figures S3–S5 in the Supplementary Material), showed that temperature was the most significant factor in optimizing hesperidin yield from peel and pomace, with 80 °C being the optimal temperature. For bagasse with liquor, which contained the highest hesperidin concentration, the optimal conditions were a temperature of 60 °C and an isothermal time of 25 min.

### 2.5. Microwave-Assisted Extraction

All methods were empirically developed and configured on the Ethos X microwave system (Milestone Srl, Sorisole, Italy) using the FastEX open vessel extraction kit. The instrument’s controller was programmed with the time and temperature parameters, and the extraction process was initiated.

The system automatically adjusted the microwave power (up to 1200 W) based on the temperature inside the vessels. The temperature was increased from room temperature to the target temperature over a 5-min ramp, then held isothermally for the specified duration. After extraction, the system underwent a 10-min cooling period. After extraction, the vessels were cooled outside the apparatus, following the manufacturer’s guidelines and the laboratory’s standard operating procedures. Each method was applied across different matrices to ensure consistent temperature conditions. Once cooled, the extracted material was transferred to 50 mL Falcon tubes. A 1.5 mL aliquot was filtered for analysis using ultra-high-performance liquid chromatography (UHPLC). The remaining material was centrifuged to separate the precipitates, while the supernatants were reduced using a speed vacuum. Finally, the aqueous content was lyophilized.

### 2.6. Characterization and Quantification of Hesperidin Samples

For hesperidin characterization, 20 mg of hesperidin was weighed and dissolved in 0.5 mL of deuterated dimethyl sulfoxide (DMSO-d6) with TMS as the internal reference. For hesperidin quantification, five samples were prepared (0.01000 ± 0.00005 g), with maleic acid (TraceCERT^®^—Sigma-Aldrich, Buchs, Switzerland) used as the internal standard. The samples were weighed using an analytical balance (SHIMADZU, Taguig, Philippines model AUW220D), and each sample was dissolved in 0.5 mL DMSO-d6.

### 2.7. NMR Analysis

NMR measurements were performed on a 14.1 Tesla (600 MHz for ^1^H) Bruker Biospin (Ettlingen, Germany) AVANCE III model spectrometer equipped with a 5 mm TCI cryoprobe. These experiments were conducted at 25 °C using D2O with TSP-d4 as the reference. For the HDO pre-saturation ^1^H spectrum, the CW pre-saturation pulse sequence (noesypr1d Bruker designation) was employed with the following parameters: acquisition time (AQ) = 3.27 s, spectral width (SW) = 18 ppm, relaxation delay (d1) = 2 s, 90° pulse (p1) = 11.50 µs, and number of scans (ns) = 64.

For the ^13^C{^1^H} qualitative experiments, the power-gated pulse sequence (zgpg30 Bruker designation) was employed with the following parameters: acquisition time (AQ) = 0.42 s, spectral width (SW) = 230 ppm, relaxation delay (d1) = 0.1 s, 90° pulse (p1) = 11 µs, and number of scans (ns) = 4096. For the semi-quantitative 13C{^1^H} analysis of the terpenoid mixture, the inverse-gated pulse sequence (zgig30 Bruker designation) was used. The acquisition parameters were set as follows: acquisition time (AQ) = 1.9 s, spectral width (SW) = 230 ppm, relaxation delay (d1) = 1 s, 90° pulse (p1) = 11 µs, and number of scans (ns) = 4096. The integrations for this analysis were performed with the same carbon type, specifically the methylene group, and as close to each other as possible.

For all heteronuclear correlation experiments (HSQC-edit and HMBC), the following parameters were applied: SWHF1 = 36,049.418 Hz, SWHF2 = 7183.908 Hz, relaxation delay (d1) = 1.0 s, number of experiments in F1 = 256, and acquisition time (AQ) in F2 = 0.27 s. Data processing was carried out using Topspin 3.5.7™ software.

Quantitative ^1^H analysis was conducted using a 9.4 Tesla (400 MHz for ^1^H) Bruker Avance III model spectrometer equipped with a 5 mm BBI probe. The continuous wave pre-saturation pulse sequence (zgpr Bruker designation) was used for HDO signal suppression with the following parameters: 90° pulse = 12.0 µs, relaxation delay (d1) = 25.0 s (greater than 7T1), acquisition time (AQ) = 4.1 s, spectral width (SW) = 18 ppm, and number of scans (ns) = 8. The purity of hesperidin was calculated from the NMR peak integrals, the initial weights of the sample and standard, the molecular masses, the number of nuclei contributing to the respective signals, and the purity of the standard. The purity of the sample was determined directly from the NMR spectrum using Equation (1):

(1) $$P_{x}=\frac{A_{x}}{A_{std}}\frac{N_{std}}{N_{x}}\frac{{MM}_{x}}{{MM}_{std}}\frac{m_{std}}{m_{x}}P_{std}$$

where A, N, MM, m, and P are integral areas, number of nuclei, molar mass, weight, and purity of analyte (x) and standard (std).

### 2.8. HPLC Analysis

HPLC-DAD analyses were conducted using an Agilent (Santa Clara, CA, USA) 1200 HPLC system with a C-18 analytical column (Phenomenex-Gemini^®^, 150 × 4.6 mm, 5 μm). Gradient elution was performed with Milli-Q HO, acetonitrile, and methanol. The chromatographic method proceeded as follows: H_2_O/CH_3_CN/CH_3_OH in the ratio 50:20:30 for 15 min, followed by 0:50:50 for 16 min, maintaining this ratio for 21 min, and then returning to the initial mobile phase composition (72:10:18) from 22 min to 34 min, with a flow rate of 1.0 mL/min. The column temperature was maintained at 30 °C, with an injection volume of 20 μL. Chromatograms were recorded using a photodiode array detector (190–600 nm) set at 285 nm. Samples derived from the orange juice industry waste were prepared in triplicate at a final concentration of 1000 µg/mL. Hesperidin quantification was performed using calibration solutions at concentrations of 0.0, 100.0, 200.0, 400.0, 600.0, 800.0, and 1000.0 μg/mL. Both SIGMA^®^ standard hesperidin and the samples were prepared in HPLC-grade CH_3_OH and subjected to ultrasonication for 10 min at 25 °C in an ultrasonic bath.

### 2.9. GC–MS Analyses

The Shimadzu (San Jose, CA, USA) AOC 5000 automated injection system (CTC Analytics 3.1.2), equipped with a Shimadzu 10 μL analytical syringe, was employed to inject 1.5 μL of samples prepared by mixing 15 μL of essential oil samples with 1.5 mL of HPLC grade hexane. This AOC system was connected to the Shimadzu (San Jose, CA, USA) QP 2010 MDGC operating in GC–MS mode (TQ8030 triple quadrupole mass spectrometer) and fitted with a DB5-MS column. The heating program followed a linear ramp at a rate of 3 °C per minute, starting at 60 °C and reaching 230 °C. Compound identification was carried out using the Robert Adams method [23].

## 3. Results and Discussion

### Traceability of Orange Waste Compounds

The collected samples were aliquoted and subjected to liquid–liquid extraction using a CDCl_3_/H_2_O (1:1 *v*/*v*) mixture to extract compounds with varying polarities, such as terpenoids and flavonoids, particularly D-limonene and hesperidin. To detect the presence of flavonoids and water-insoluble carbohydrates, the material was lyophilized and then resuspended in DMSO-d6, as outlined in the detailed flowchart in the Supplementary Material (Figure S1).

The NMR experiments identified key compounds, including D-limonene, myrcene, linalool, pinene, hesperidin, and carbohydrates. These findings provide valuable insights into the chemical composition of orange juice waste during the re-extraction process, highlighting its significance for effective waste management and potential value-added applications.

**Figure 2:**
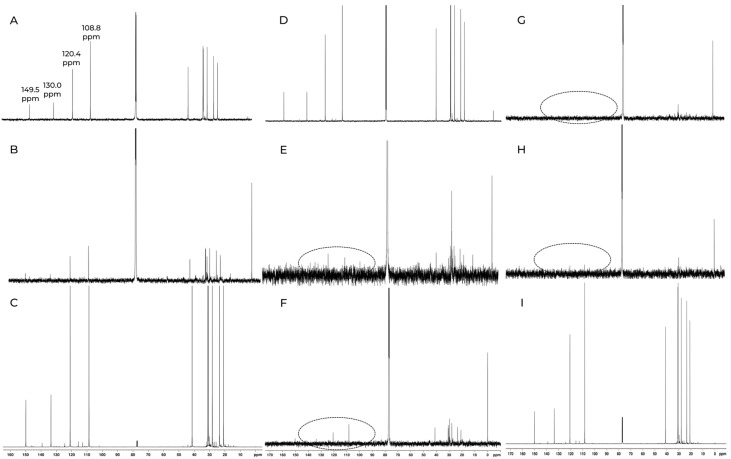
^13^C {^1^H} spectrum acquired in a Bruker 14.1 T (600 MHz for ^1^H) with a TCI cryoprobe of CDCl_3_-extracted samples from various stages of the orange bagasse processing. (**A**) Concentrator inlet; (**B**) Concentrator outlet; (**C**) Polishing outlet; (**D**) D-oiler inlet; (**E**) D-oiler outlet; (**F**) Lagoon inlet; (**G**) Lagoon interior; (**H**) Lagoon outlet; (**I**) Lagoon “sludge”.

Figure 2 illustrates the traceability of the major terpenoid compound, D-limonene, throughout the industrial chain of bagasse processing, as depicted by the 13C NMR spectra (δ 149.5, 130.0, 120.4, and 108.8 ppm). The unambiguous assignment of the signals is provided in the Supplementary Material [24]. The figure also highlights the process stages where the highest concentration of D-limonene is observed, with a significant reduction occurring after the extraction steps, particularly during the de-oiling phase. Despite the high efficiency in separating and recovering terpenoid compounds, D-limonene was still detectable in the sedimentation pond, where process waste accumulates.

Analyzing Figure 2F–I in relation to the spectra of the aqueous and colloidal contents of the fermentation pond reveals that intense D-limonene signals appear only in the colloidal material. This confirms that, as expected, D-limonene does not dissolve well in water, leading to changes in its physicochemical properties. However, the high concentration of colloidal material in the treatment lagoon contained a significant amount of D-limonene, likely absorbed by the abundant polysaccharides present in this material.

**Figure 3:**
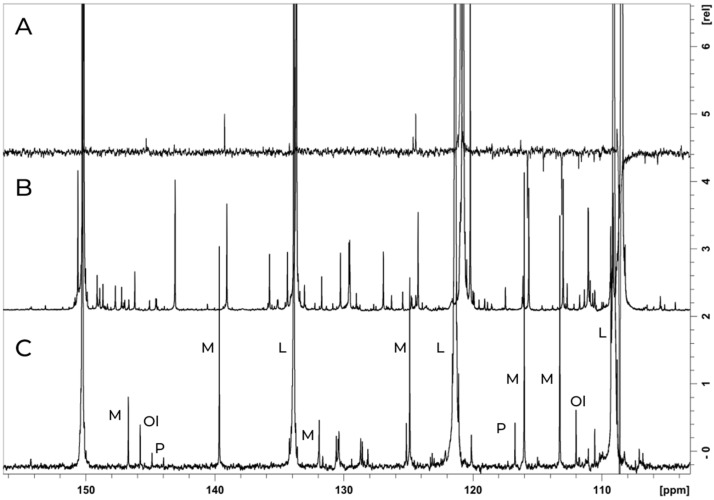
^13^C {^1^H} spectrum obtained from a Bruker 14.1 T (600 MHz for ^1^H) with a TCI cryoprobe of the polish machine sample extracted with CDCl_3_ (**C**), overlaid with the spectrum of valencene (**B**) and the DEPT 135 spectrum of the polisher machine sample (**A**). Key compounds, such as D-limonene (L), myrcene (M), linalool (Ol), and pinene (P), are labeled for reference.

In addition to D-limonene, the presence of minor terpenoid compounds was tracked throughout the bagasse re-extraction process and semi-quantified relative to the major compound. Characteristic chemical shift signals for terpenes, including D-limonene (L), myrcene (M), linalool (Ol), and pinene (P), are clearly observed in the ^13^C spectrum of the biomass extracted from the polisher machine (Figure 3). These assignments align with the composition of pure essential oil fractions, as determined by the GC–MS technique.

Semi-quantification of myrcene relative to D-limonene (L) signals in ^13^C at δ 124.2 ppm (CH_2_) and 120.8 ppm (CH_2_), respectively, resulted in 1.9% of the compound. Similarly, for linalool (OI), integration of signals from quaternary carbons at δ 73.5 and 150.1 ppm yielded a concentration of approximately 0.4%. Pinene (P) was semi-quantified at approximately 0.3% by integrating signals at δ 38.4 and 150.1 ppm, corresponding to quaternary carbons of pinene and D-limonene.

Although these relative quantifications do not provide absolute value, they offer a fast and efficient way to track the compositional abundance of samples throughout the process without requiring separation, purification, or concentration steps. Traditional and semi-industrial methods for extracting essential oils typically involve cold pressing and distillation. In cold pressing, oils are mechanically extracted from the fruit peel and cuticle, producing an aqueous emulsion. This emulsion then undergoes centrifugation and distillation to recover the essential oils [7]. Inefficiencies in these steps can alter the composition of the oils, potentially impacting their quality, authenticity, and market value.

This process results in five distinct phases: citrus terpene, oil phase, cold-pressed orange oil, valencene, and valencene-rich fraction. The ^13^C NMR spectra indicates a predominance of D-limonene across these phases, with valencene being a minority compound primarily found in the valencene-rich fraction. Moreover, myrcene, α-pinene, and linalool are detected in all phases. According to the literature [25], the ^13^C NMR chemical shifts for myrcene, linalool, and pinene are at δ 116 ppm (CH2), 113 ppm (CH_2_), 124 ppm (CH), 131 ppm (C-quaternary); δ 115 ppm (CH2), 145.8 ppm (CH), 73 ppm (C-quaternary), 124.9 ppm (CH), 131 ppm (C-quaternary); and δ 144.3 ppm (C-quaternary), 116 ppm (CH), 38 ppm (C-quaternary), respectively. Figure S6 in the Supplementary Material presents the gas chromatography–mass spectrometry (GC–MS) analysis, identifying 11 compounds in the valencene phase and six compounds in other phases. These findings show a strong correlation with the NMR spectra, except for sabinene and ocimene, which are present in concentrations lower than 0.5% and 0.2%, respectively. This highlights the effectiveness of NMR in analyzing complex mixtures without requiring isolation steps [22]. The 11 terpenes detected by GC–MS are listed and numbered according to the data presented in Table 2.

During the concentration, ultracentrifugation, and distillation steps of the wastes from orange juice extraction, the presence of hesperidin was identified by ^13^C NMR spectra. Key peaks at 196.9, 165.1, 162.4, 147.9, 146.4, 130.9, 117.9, 114.1, and 112.0 ppm were used as target signals for its detection in the matrices. Hesperidin was observed in the concentration steps immediately after the concentrator and polisher (Figure S7 in the Supplementary Material). However, the compound was not detected in the subsequent steps, as shown in Figures S8 and S9 in the Supplementary Material. During the concentration, ultracentrifugation, and distillation steps of orange juice extraction waste, the presence of hesperidin was identified through ^13^C NMR spectroscopy. Key peaks at 196.9, 165.1, 162.4, 147.9, 146.4, 130.9, 117.9, 114.1, and 112.0 ppm served as target signals for its detection in the matrices. Hesperidin was observed during the concentration steps, specifically immediately after the concentrator and polisher (Figure S7, Supplementary Material). However, it was not detected in the subsequent stages, as shown in Figures S8 and S9.

Additional spectral information and chemical shift tables for major identified compounds—including valencene, D-limonene, and hesperidin—are provided in the Supplementary Material. As noted in [26], the structural elucidation of glycosylated flavonoids relies heavily on ^13^C, HSQC, and HMBC NMR spectra, which provide essential insights into the number, nature, position, and conformation of sugar moieties within the molecule.

Analysis of the ^13^C, HSQC, and HMBC spectra of hesperidin confirmed its structural identity, and key signals indicative of the flavanone skeleton were identified, including C2/H2 at δ 5.5/78.3 (dd J = 12.30 3.26Hz), representing three hydrogens of the 1,3,4-trisubstituted ring, and δ 6.90 (dd = 8.39 2.00 Hz), 6.94 (dd = 8.39 2.00 Hz), and 6.95 (d J = 2.00 Hz) for the two anomeric hydrogens and carbons of glucose and rhamnose at δ 99.4/4.97 (1″CH) and 100.6/4.53 (1‴CH), respectively [26].

Hesperidin was selected as the focal point of this study due to its significant operational challenges in the juice industry, particularly in production lines. Its presence is considered undesirable because of its unpleasant sensory properties, which negatively affect orange juice quality. Additionally, hesperidin contributes to blockages, equipment malfunctions in unit operations, and disruptions in transport processes within industrial facilities. Despite these challenges, the global hesperidin market was valued at approximately USD 81 million in 2019, with projections indicating growth to USD 125.2 million by 2026, underscoring its economic significance [27]. For this study and based on the experimental design, bagasse with liquor was chosen as the starting material for new extraction processes. Hesperidin quantification was conducted using UHPLC-DAD, while ¹H and ^13^C NMR analyses were performed on concentrated and lyophilized samples. The NMR spectra confirmed that the selected solvent strategy (80:20 ethanol/water) effectively extracted flavonoid compounds, as evidenced by UHPLC analysis, along with polysaccharides such as pectin. To support compositional profiling, Figure S10 in the Supplementary Material presents the analytical signals of various extracts (bagasse with liquor, bagasse, and peel) compared to standard spectra of hesperidin, pectin, and essential oil components (terpenes: valencene and D-limonene).

**Figure 4:**
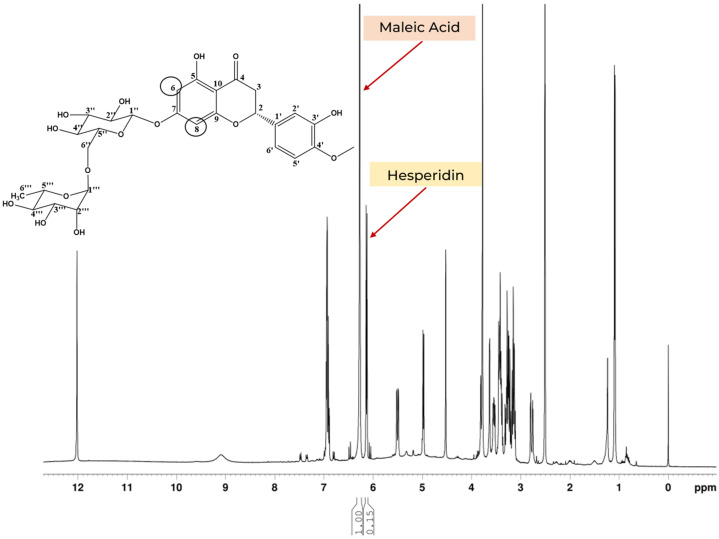
Quantification ^1^H NMR of hesperidin in DMSO-d_6_, using a Bruker 9.4T (400 MHz for ^1^H) with a BBI probe at 25 °C. Maleic acid—TraceCERT^®^ was used as an internal standard in this analysis.

Quantitative ^1^H NMR experiments were conducted in quintuplicate for hesperidin, integrating the signal at 6.14 ppm and referencing it against maleic acid at 6.13 ppm for both corresponding CH_2_ groups (Figure 4). The average purity of hesperidin, determined by quantitative NMR, was 87.66% ± 0.99 (Table 3). Additionally, purity determination analyses by HPLC with DAD detection were performed in triplicate, resulting in an analytical curve (Figure S11, Supplementary Material). The chromatographic assay yielded a value of 84.30%, closely matching the qNMR result (Table 4). However, it is important to note that the chromatographic assay required significantly longer analysis times and higher material consumption [28].

## 4. Conclusions

According to all the results presented in this study, analyzing all the stages of the citrus waste plant led to a rich understanding of the process and compounds present in each stage and helped the industry, the environment, and science to propose and implement improvements for better management of the industry process.

Therefore, the present study demonstrated the effectiveness of green sample preparation methods and the analytical capabilities of NMR for analyzing mixtures in complex matrices. This was achieved by tracing D-limonene and hesperidin throughout the orange bagasse re-extraction process, as well as the processing of wastes. Similar observations were made for the major constituents in the “oil phase” used by industrial plants. These findings support the application of NMR for monitoring chemical composition at all stages of industrial citrus processing.

The analysis of the content after essential oil removal showed no detectable hesperidin, suggesting that the flavonoid is adsorbed onto the pectic and cellulosic components of the biomass, which are eventually processed into bran. This underscores the potential of NMR for chemical traceability, enabling the identification of different production stages where both target and non-target compounds can be detected and characterized.

Furthermore, the assessment of hesperidin purity underscores the industry’s commitment to recovering this valuable bioactive compound. This not only supports the principles of a circular economy but also helps reduce the environmental impact associated with its disposal.

In this context, nuclear magnetic resonance (NMR) has proven to be a powerful tool for tracking major products at key stages of the process and semi-quantifying compounds. By employing simple liquid–liquid extraction techniques, NMR enables the analysis of complex mixtures within the matrices. Additionally, microwave-assisted extraction has emerged as an efficient and eco-friendly method for extracting hesperidin and other bioactive compounds. This technique yields high-purity hesperidin, as confirmed by both NMR (87.66%) and HPLC (84.30%) analyses, along with other valuable compounds such as α-pinene, sabinene, β-myrcene, β-ocimene, D-limonene, linalool, copaene, β-guaiane, valencene, and azulene.

The reuse and valorization of citrus peel waste could play a crucial role in reducing environmental impact. By transforming this waste into value-added products, industries can contribute to the development of functional foods, cosmetics, and preventive therapies for specific diseases. This approach not only enhances the sustainability of citrus processing but also creates new opportunities for businesses in the sector.

## Figures and Tables

**Table 1:** Hesperidin concentration achieved through the experimental design using UHPLC-PDA.

Samples	Sample Name	Concentration (mg/L)	Concentration (g/kg)
Pomace	OP1 B 1	203.24	1.02
	OP1 B 2	102.97	4.12
	OP1 B 3	287.82	1.44
	OP1 B 4	115.93	4.64
	OP1 B 5	71.76	2.87
	OP1 B 6	90.85	3.63
	OP1 B 7	74.37	2.97
Bagasse with liquor	OP1 BL 1	54.37	2.17
	OP1 BL 2	184.85	7.39
	OP1 BL 3	60.08	2.40
	OP1 BL 4	159.79	6.39
	OP1 BL 5	186.51	7.46
	OP1 BL 6	194.13	7.77
	OP1 BL 7	197.61	7.90
Peel	OP1 P 1	172.76	1.73
	OP1 P 2	170.60	6.82
	OP1 P 3	283.73	1.42
	OP1 P 4	189.73	7.59
	OP1 P 5	99.89	4.00
	OP1 P 6	149.74	5.99
	OP1 P 7	134.39	5.38

Data: Samples OP = oil phase; B = pomace; BL = bagasse with licor; P = peel; digits (1–7) = replicates.

**Table 2:** Terpenes identified in samples. Retention time registered in column 2, percentage of total integrated area in columns 3, 4, 5, 6, and 7, the percentage of a match using library spectra in column 8, and the relative retention index in column 8.

Peak	Rt	Sample 1	Sample 2	Sample 3	Sample 4	Sample 5	Library %
1	4.68	0.89	0.80	0.76	0.73	0.72	α-pinene (97)
2	5.63	0.55	0.49	0.66	0.79	0.16	Sabinene (95)
3	6.05	1.72	1.56	1.47	1.39	1.36	β-myrcene (96)
4	6.64	0.11	0.21	0.27	0.15	0.16	β-ocimene (95)
5	7.39	95.85	96.13	96.35	96.27	91.73	*D-limonene* (96)
6	9.70	0.78	0.60	0.49	0.67	0.09	Linalol (93)
7	21.04	-	-	-	-	0.18	Copaene (94)
8	25.41	-	-	-	-	0.24	β-guaiane (90)
9	25.76	-	-	-	-	4.61	(+) Valencene (94)
10	25.89	-	-	-	-	0.33	Azulene (90)
11	26.80	-	-	-	-	0.42	Selinene (90)

Data: Samples 1 = Oil phase; 2 = Cold Pressed; 3 = Citric Terpene; 4 = Orange oil; 5 = Valencene phase.

**Table 3:** Quantification ^1^H NMR of hesperidin in DMSO-d_6_, using a Bruker 9.4T (400 MHz for ^1^H) with a BBI probe at 25 °C.

Analyte/Internal Standard	Mass (g)	Purity (%)
1 = Hesperidin/Maleic acid	0.01005/0.01035	86.490
2 = Hesperidin/Maleic acid	0.01006/0.01016	86.522
3 = Hesperidin/Maleic acid	0.01005/0.01010	89.334
4 = Hesperidin/Maleic acid	0.01002/0.01007	87.511
5 = Hesperidin/Maleic acid	0.01002/0.01003	88.457
Average		87.663

Data: Molar mass of hesperidin = 610.565 g·mol^−1^; Integration signal = δ 6.13 H8 (average point 6.13–6.14) corresponding to 2 H.

**Table 4:** Results of the hesperidin quantification in samples from the orange juice industry waste.

Quantification of Hesperidin in the Samples of Citrus Juice Waste						
	1	2	3	Average	Concentration (µg·mL^−1^)	Concentration (%)
Sample	906.03	996.49	929.05	943.86	843.04	84.30

## Data Availability

The original contributions presented in the study are included in the article/Supplementary Material, further inquiries can be directed to the corresponding author.

## Supplementary Materials

The following supporting information can be downloaded at https://www.mdpi.com/article/10.3390/foods14040642/s1, Figure S1. Flow chart of the sample preparation for the NMR analysis; Figure S2. Plot of marginal means (A), pareto chart (B) contour curves chart (C), and response surface (D) peel; Figure S3. Plot of marginal means (A), pareto chart (B), contour curves chart (C), and response surface (D) bagasse with liquor; Figure S4. Plot of marginal means (A), pareto chart (B), contour curves chart (C), and response surface (D) pomace; Figure S5. Calibration curve for hesperidin quantification; Figure S6. Chromatogram of the valencene phase; Figure S7. ^13^C {^1^H} spectra obtained with a Bruker 14.1 T (600 MHz for ^1^H) with a TCI cryoprobe of the lyophilized samples from the re-extraction of orange waste in DMSO-d6; Figure S8. ^13^C {^1^H} spectra obtained with a Bruker 14.1 T (600 MHz for ^1^H) with a TCI cryoprobe of the lyophilized samples from the re-extraction of orange waste in DMSO-d6; Figure S9. ^13^C {^1^H} spectra obtained with a Bruker 14.1 T (600 MHz for ^1^H) with a TCI cryoprobe of the lyophilized samples from the re-extraction of orange waste in DMSO-d6; Figure S10. ^13^C {^1^H} spectra obtained with a Bruker 14.1 T (600 MHz for ^1^H) with a TCI cryoprobe of Pomace (BA-blue); bagasse with liquor (BL-red); peel (CA-green); hesperidin (purple); pectin (yellow) and terpenes—valencene and D-limonene (orange); Figure S11. Calibration curve and the chromatograms of the hesperidin standard and the samples from orange juice industry waste; Figure S12. ^13^C NMR spectra of the oil fraction rich in valencene, labeled “Valencene” CDCl_3_, 14.1 Tesla at 25 °C; Figure S13. HSQC contour map of the oil fraction rich in d-limonene in CDCl_3_, 14.1 Tesla at 25 °C; Figure S14. ^13^C NMR spectrum of the hesperidin in DMSO-d6, 14.1 Tesla at 25 °C; Figure S15. HSQC contour map of the hesperidin in DMSO-d6, 14.1 Tesla at 25 °C; Figure S16. HMBC contour map of the hesperidin in DMSO-d6, 14.1 Tesla at 25 °C; Table S1. Statistical planning for microwave-assisted extraction of hesperidin; Table S2. Chemical shift (δ ppm) of the valencene in CDCl_3_, 14.1 T at 25 °C; Table S3. Chemical shift data (δ ppm) of the d-limonene in CDCl_3_, 14.1 T at 25 °C; Table S4. Chemical shift (δ ppm) of the hesperidin in DMSO-d6, 14.1 T at 25 °C.
