# Supplementary material for: Green Extraction and NMR Analysis of Bioactives from Orange Juice Waste
Source: Foods. 2025 Feb 14;14(4):642. doi: 10.3390/foods14040642 (PMC11854020; doi:10.3390/foods14040642)
Supplement: Supplementary file 1 [file foods-14-00642-s001.zip › foods-3447208-supplementary.pdf]

Supplementary Materials

GREEN EXTRACTION AND NMR ANALYSIS OF BIOACTIVES FROM ORANGE JUICE WASTE

Paula Scarabotto Penteado[1*]; Maria Carolina B. Di-Medeiros Leal[1*]; Maria Gabriela Aparecida Carosio[2]; Alef dos Santos[1]; Mateus Lodi Segatto[1], Daniel Petinatti Pavarini[3]; Danielle Fernandes da Silva[1]; Jéssica Cristina Amaral[1]; Maria Fátima G. F. da Silva[1]; Vânia G. Zuin Zeidler[4]; Antonio Gilberto Ferreira[1]

[1] Department of Chemistry, Federal University of São Carlos, SP, Brazil

[2] Department of Chemistry, University of Wisconsin-Madison, WI, USA

[3] Perdue Animal Nutrition, Salisbury, MD, USA

[4] Institute of Sustainable Chemistry, Leuphana University Lüneburg, NI, Germany

* Correspondence: p.scarabotto20@gmail.com; caroldimedeiros@gmail.com

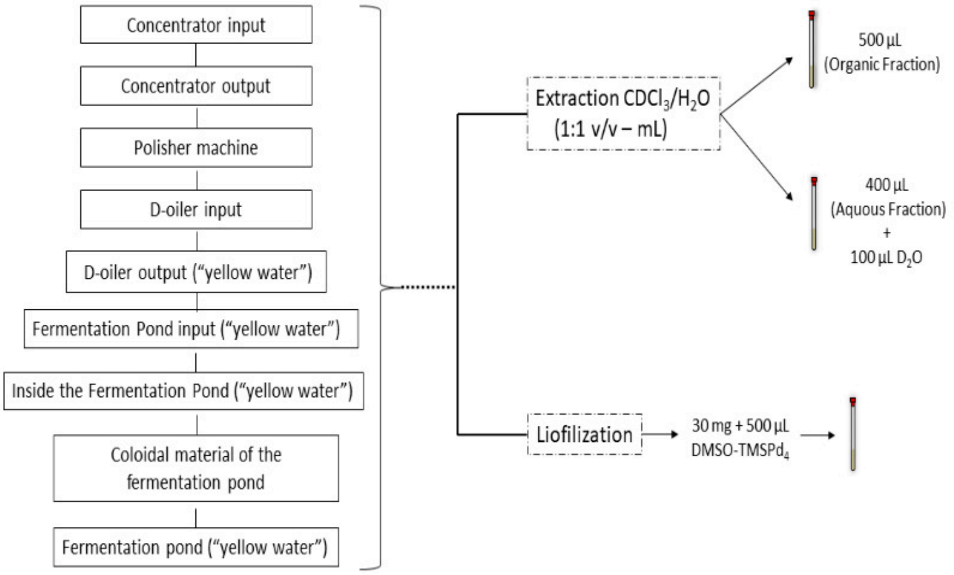

Figure S1. Flow chart of the sample preparation for the NMR analysis.

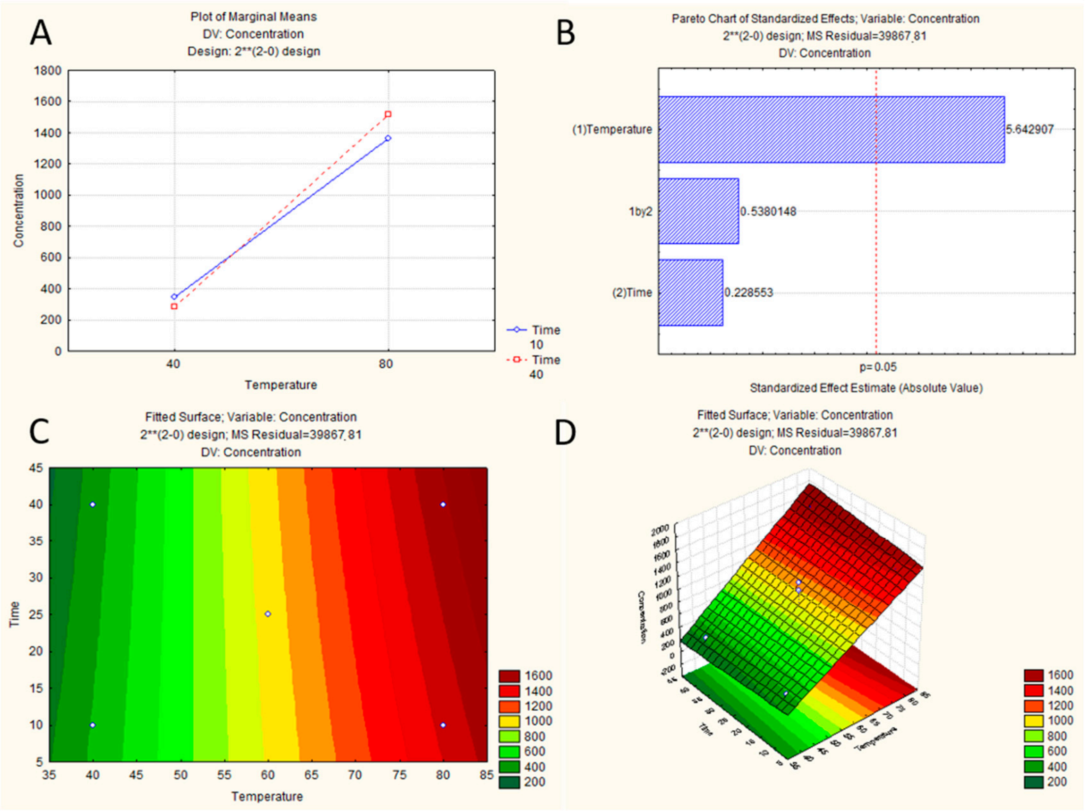

Figure S2. Plot of marginal means (A), pareto chart (B) contour curves chart (C) and response surface (D) – peel.

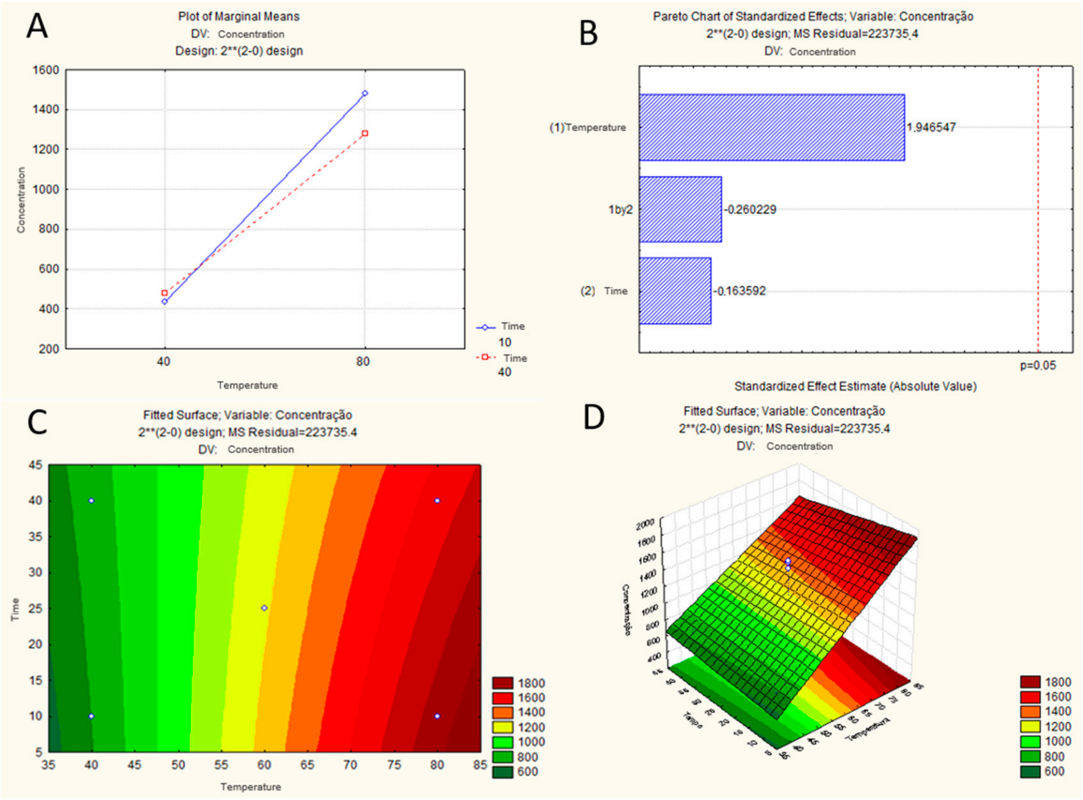

Figure S3. Plot of marginal means (A), pareto chart (B), contour curves chart (C) and response surface (D) – bagasse with liquor.

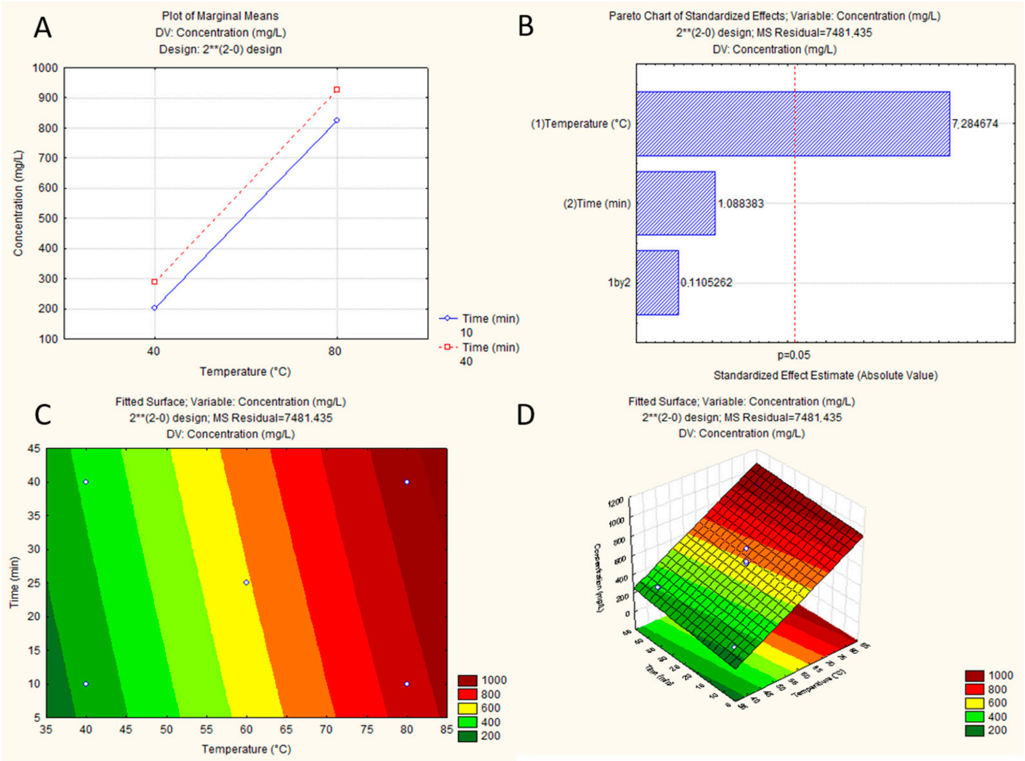

Figure S4. Plot of marginal means (A), pareto chart (B), contour curves chart (C) and response surface (D) – pomace.

SampleName	Injection	Name	RT	Area	Average Area (uV*sec)	Var	Error	Error %	Concentration (mg/L)
CC Hesp Point 7	3	Hesperidin	5.631	1818820	**1758500**	3117035146	55830	3%	**404**
	1	Hesperidin	5.575	1748043					
	2	Hesperidin	5.561	1708638					
CC Hesp Point 6	3	Hesperidin	5.57	1329451	**1325225**	13707907	3702	0%	**303**
	2	Hesperidin	5.603	1323672					
	1	Hesperidin	5.639	1322552					
CC Hesp Point 5	3	Hesperidin	5.64	876659	**876111**	266844	517	0%	**202**
	2	Hesperidin	5.581	875633					
	1	Hesperidin	5.602	876041					
CC Hesp Point 4	3	Hesperidin	5.643	436115	**427769**	52687520	7259	2%	**101**
	2	Hesperidin	5.596	422931					
	1	Hesperidin	5.627	424260					
CC Hesp Point 3	3	Hesperidin	5.577	248382	**239736**	72410556	8509	4%	**61**
	2	Hesperidin	5.619	239457					
	1	Hesperidin	5.633	231370					
CC Hesp Point 2	3	Hesperidin	5.584	64562	**63187**	2736560	1654	3%	**20**
	2	Hesperidin	5.597	63647					
	1	Hesperidin	5.588	61351					
CC Hesp Point 1	3	Hesperidin	5.594	29604	**29883**	210091	458	2%	**10**
	2	Hesperidin	5.631	29633					
	1	Hesperidin	5.723	30412					

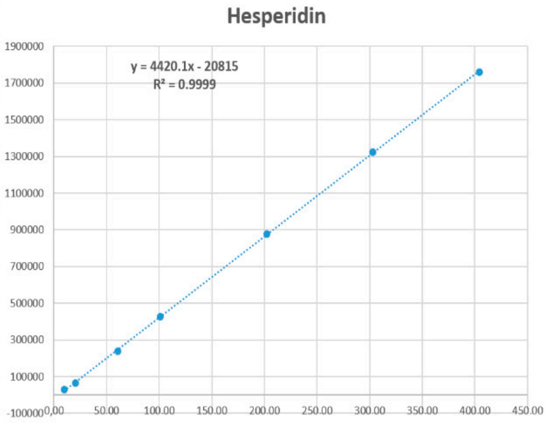

Figure S5. Calibration curve for hesperidin quantification.

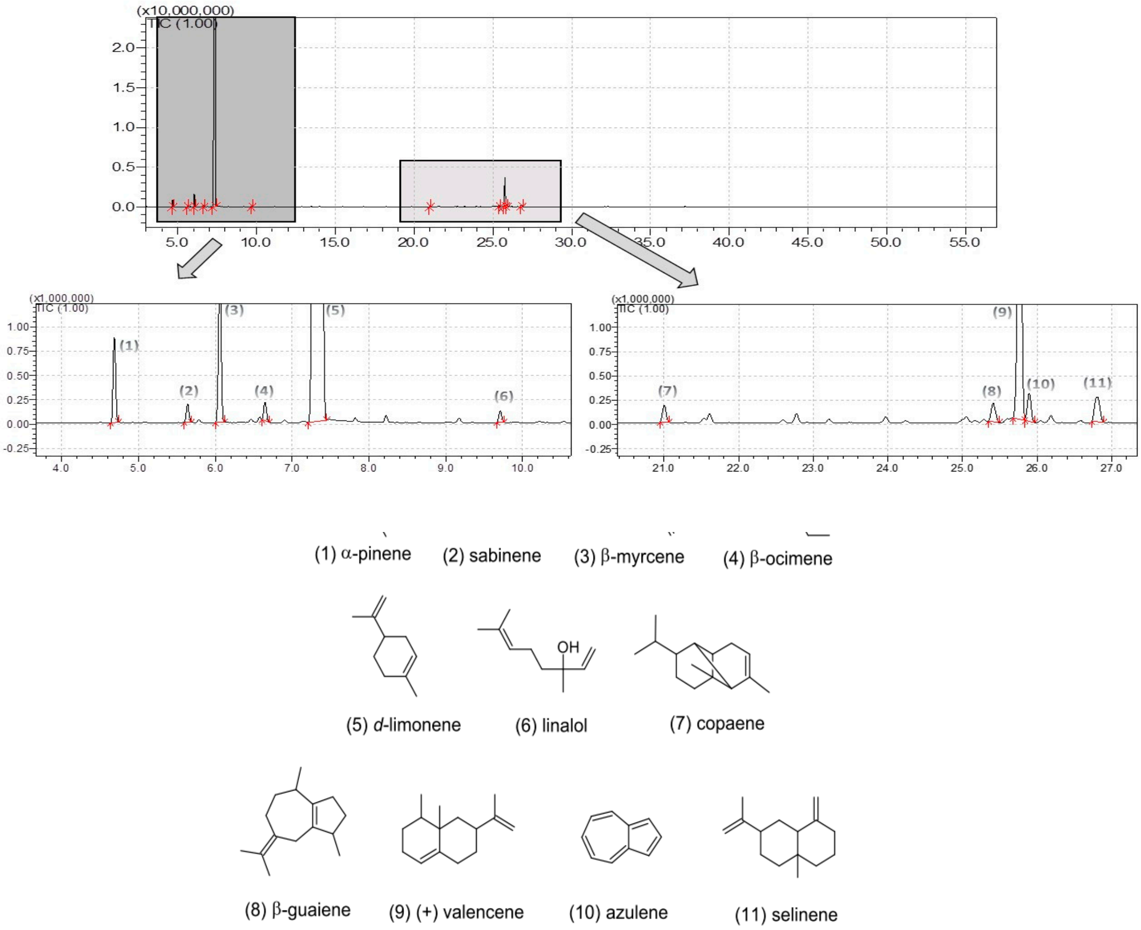

Figure S6. Chromatogram of the valencene phase.

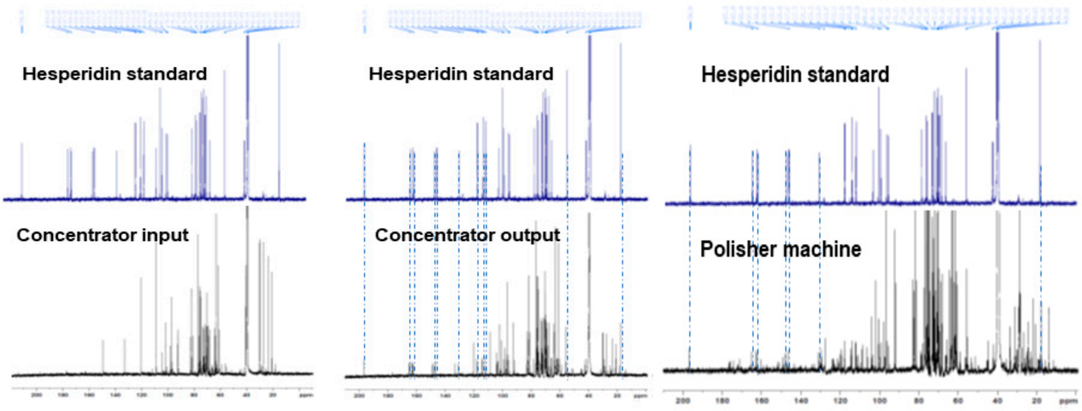

Figure S7. ^{13}C $\{^1H\}$ spectra obtained with a Bruker 14.1 T (600 MHz for ^1H) with a TCI cryoprobe of the lyophilized samples from the re-extraction of orange waste in DMSO-d6.

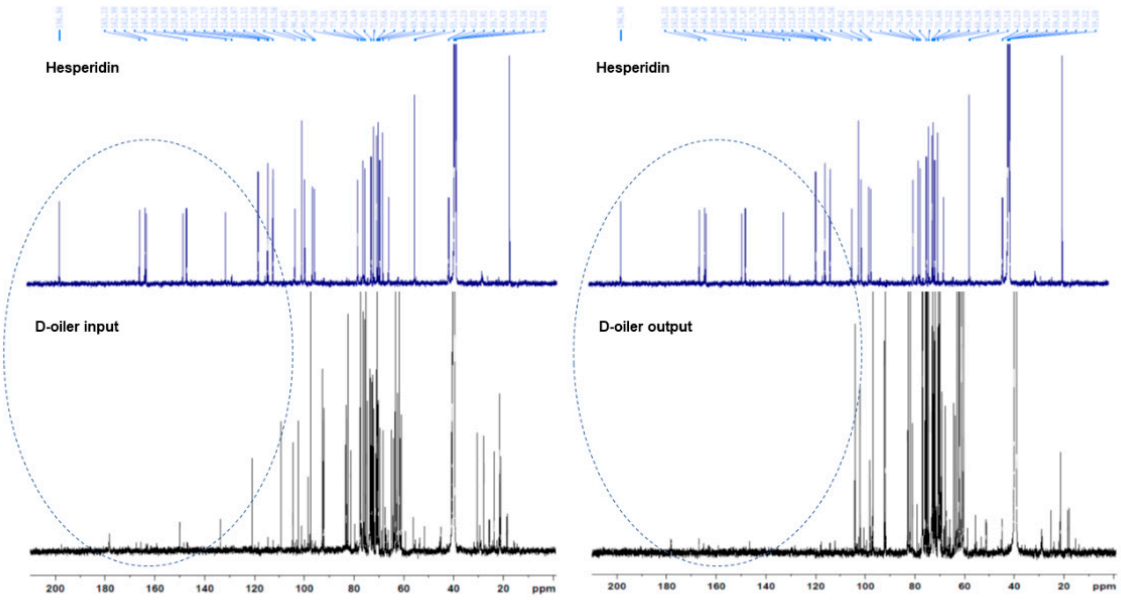

Figure S8. ^{13}C {^{1}H} spectra obtained with a Bruker 14.1 T (600 MHz for ^{1}H) with a TCI cryoprobe of the lyophilized samples from the re-extraction of orange waste in DMSO-d6.

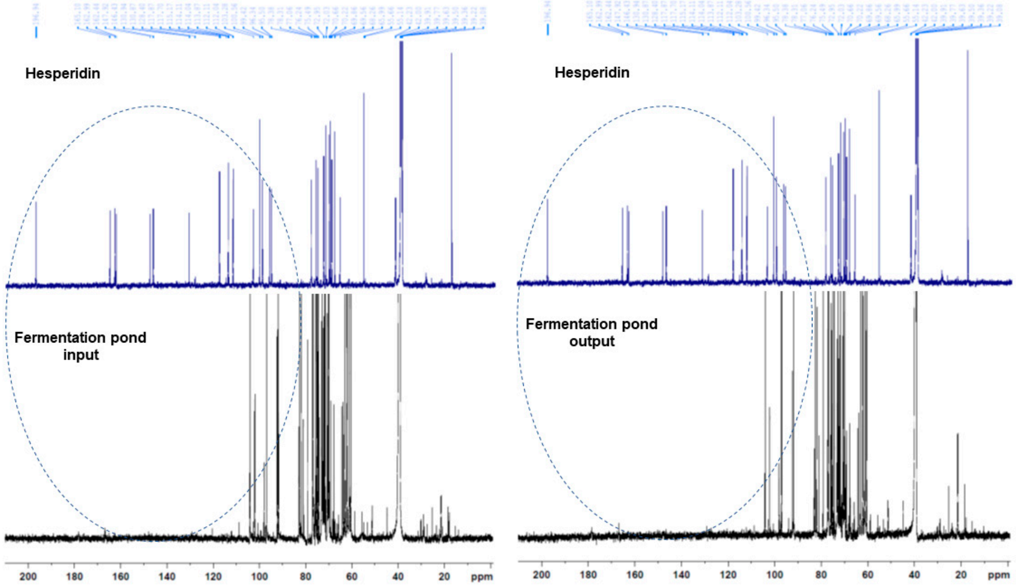

Figure S9. ^{13}C {^{1}H} spectra obtained with a Bruker 14.1 T (600 MHz for ^{1}H) with a TCI cryoprobe of the lyophilized samples from the re-extraction of orange waste in DMSO-d6.

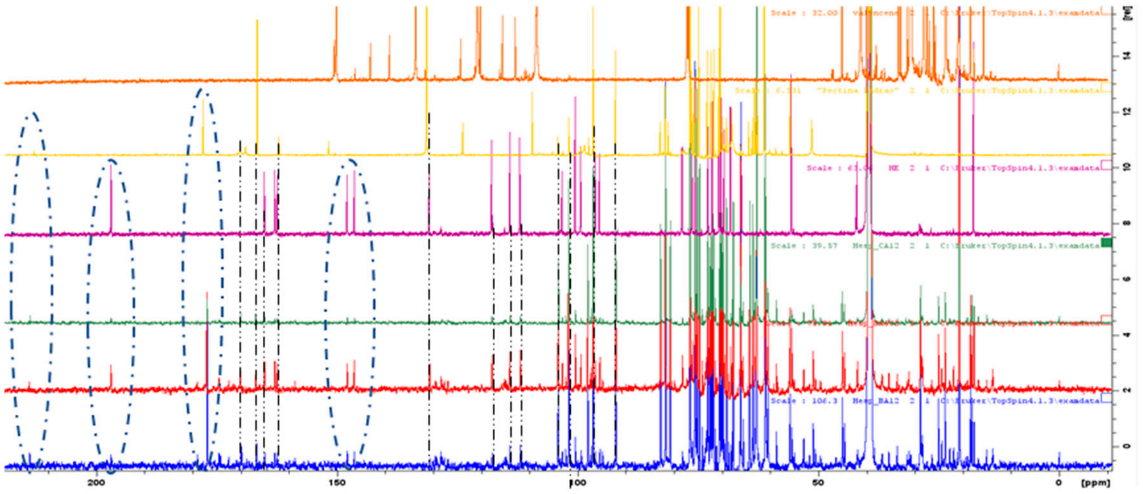

Figure S10. ^{13}C {^{1}H} spectra obtained with a Bruker 14.1 T (600 MHz for ^{1}H) with a TCI cryoprobe of Pomace (BA-blue); bagasse with liquor (BL-red); peel (CA-green); hesperidin (purple); pectin (yellow) and terpenes – valencene and D-limonene (orange).

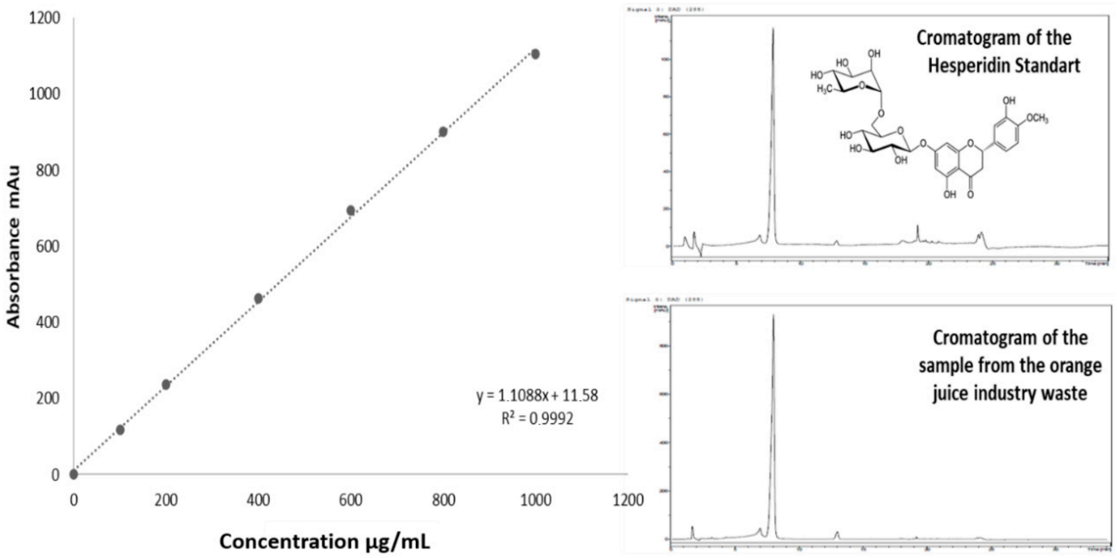

Figure S11. Calibration curve and the chromatograms of the hesperidin standard and the samples from orange juice industry waste.

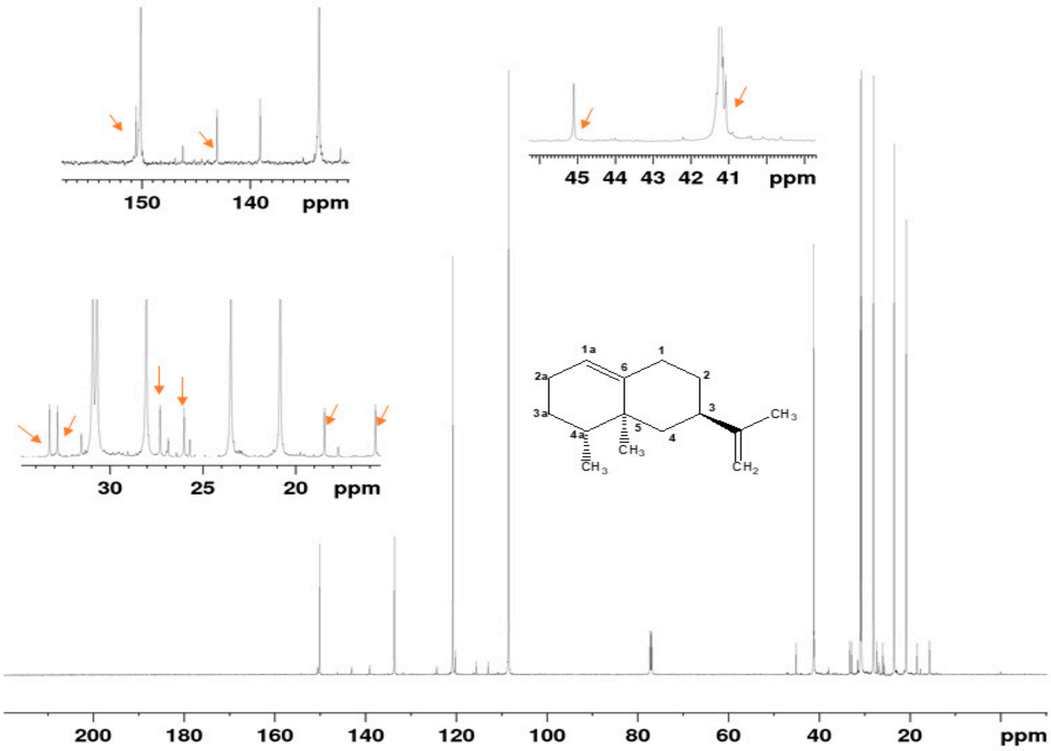

Figure S12. ^{13}C NMR spectra of the oil fraction rich in valencene, labeled "Valencene" $CDCl_3$, 14.1 Tesla at 25°C.

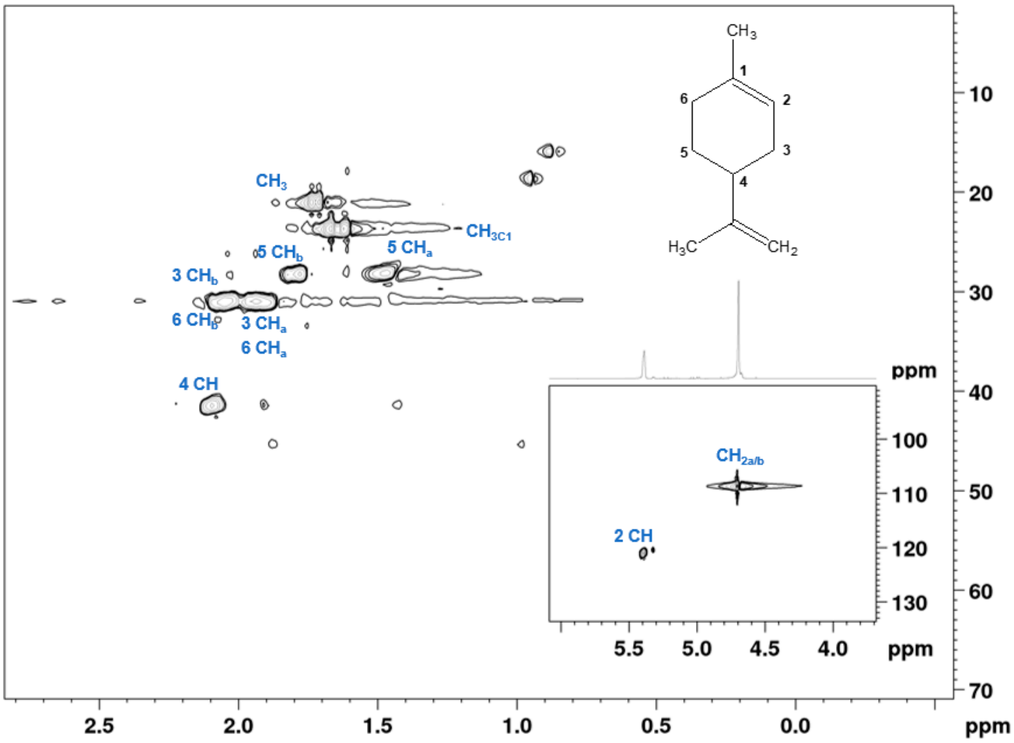

Figure S13. HSQC contour map of the oil fraction rich in d-limonene in $CDCl_3$, 14.1 Tesla at 25 °C

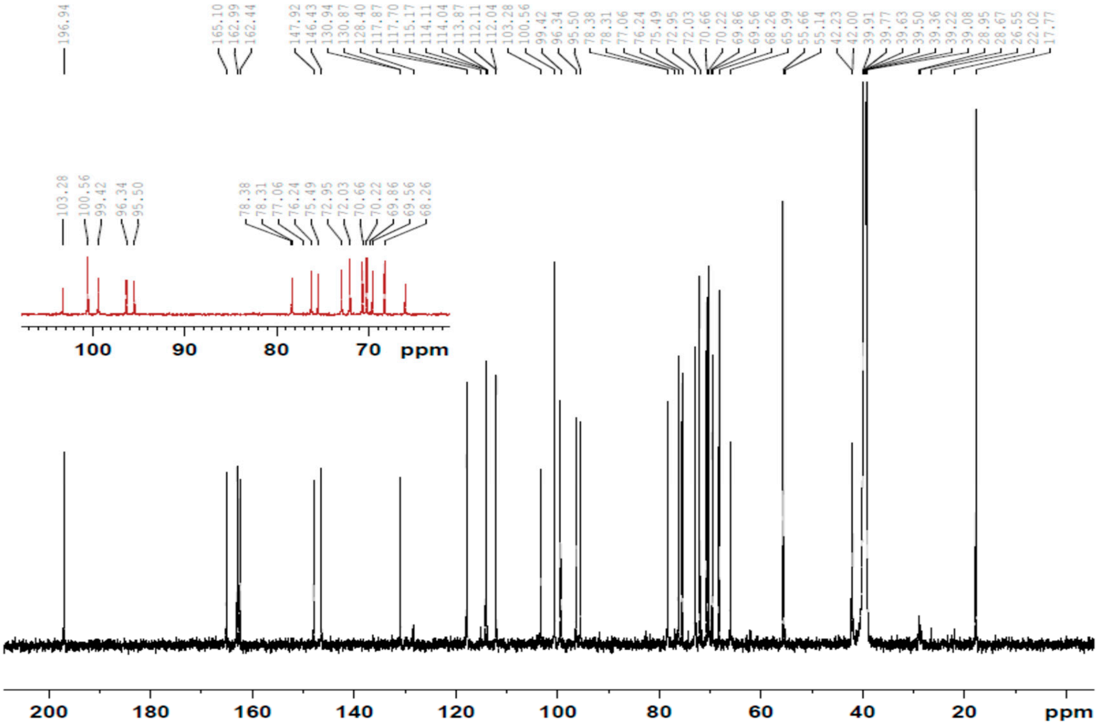

Figure S14. ^{13}C NMR spectrum of the hesperidin in DMSO-d6, 14.1 Tesla at 25°C.

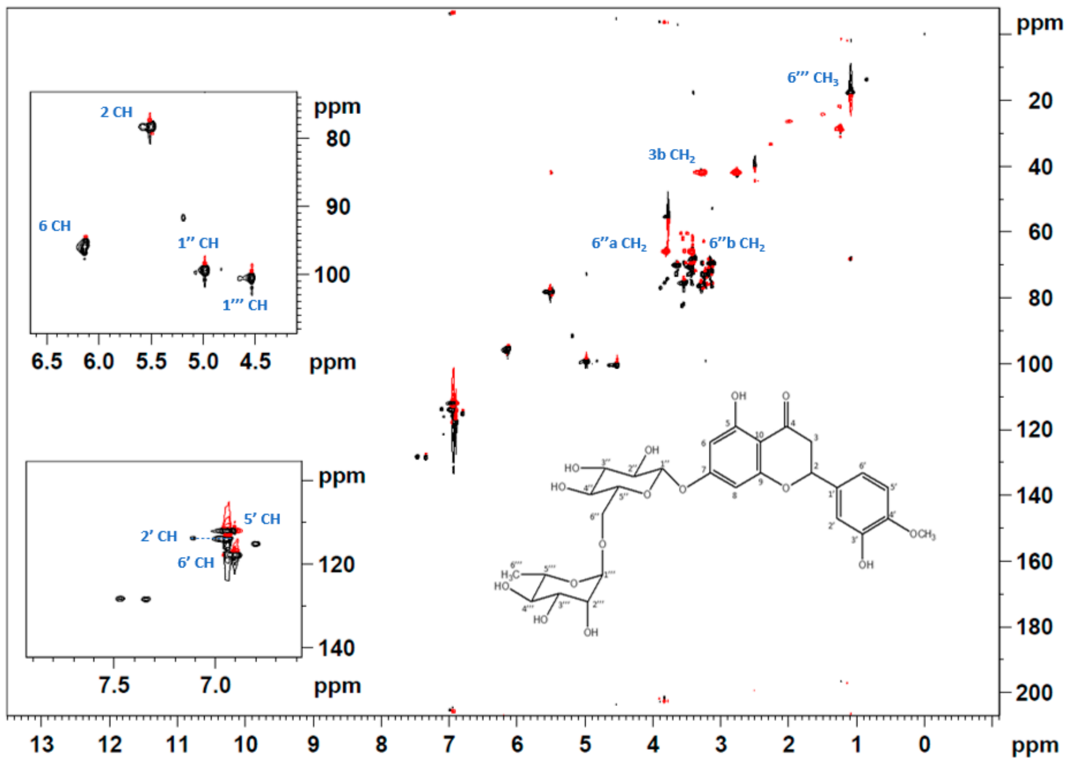

Figure S15. HSQC contour map of the hesperidin in DMSO-d6, 14.1 Tesla at 25°C.

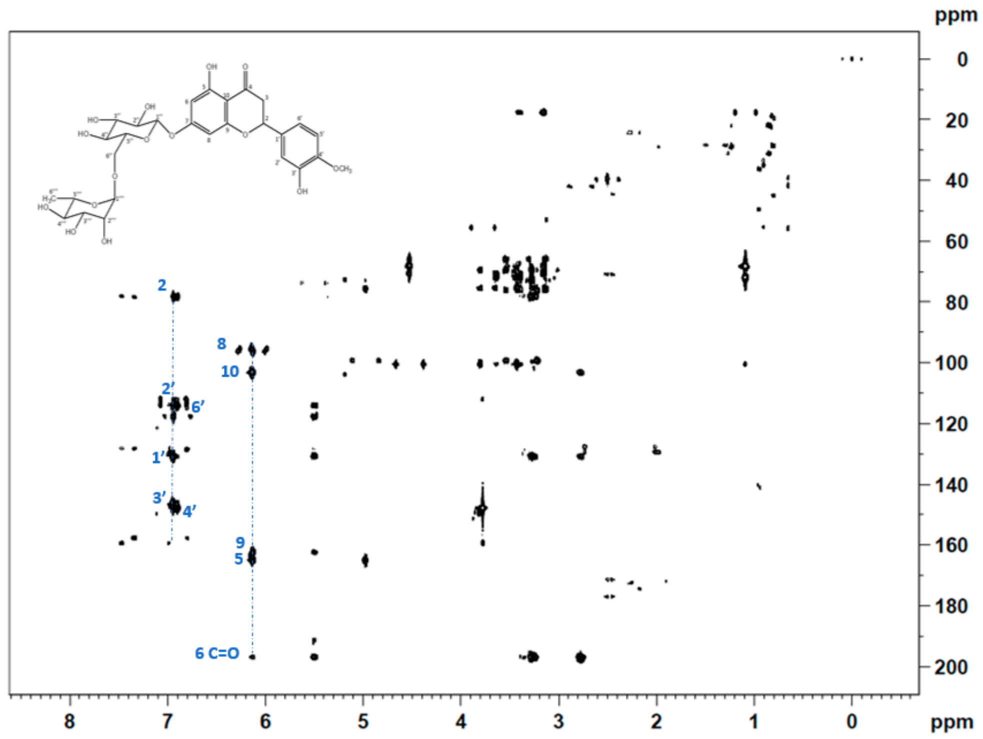

Figure S16. HMBC contour map of the hesperidin in DMSO-d6, 14.1 Tesla at 25°C.

Table S1. Statistical planning for microwave-assisted extraction of hesperidin.

Samples	Temperature (ºC)	Isothermal time (minutes)
2	80	10
4	80	40
5 C	60	25
3	40	40
6 C	60	25
7 C	60	25
1	40	10

Table S2. Chemical shift (δ ppm) of the valencene in $CDCl_3$, 14.1 T at 25 ºC.

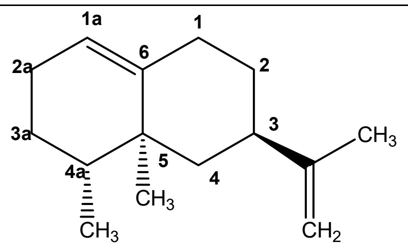

N°	ACD ($^1H/^{13}C$)	δ ^1H Multiplicity	δ ^{13}C	HMBC
1	2.24;2.27;2.09/**31.45**	2.31 m	**33.3**	
2	1.83;1.35;1.33/**30.85**	1.72 m	**32.8**	32.8; 41.0; 108; 150
3	5.53/**39.0**		**41.0**	
4	1.85;1.48/**42.45**	2.08 m	**45.0**	
5	----/**37.95**	----	**38.0**	---
6	----/**143.40**	----	**143.0**	---
1a	5.34/**120.30**	5.14 t	**120.0**	15.8; 25.8
2a	2.04;2.07;2.09;2.12/**27.25**	2.05 m	**25.8**	
3a	1.35;1.33;1.27/**39.80**		**27.2**	
4a	------		**41.0**	
4a-CH_3	0.88/**15.85**	0.87 sl	**15.8**	
5-CH_3	0.98/**19.90**	0.94 sl	**19.9**	
3-C=	----/**150.20**	---	**150.0**	----
3-CH_2	4.69/**108.25**	4.67 m	**108.0**	
3-CH_3	1.71/**20.55**	1.63 sl	**20.5**	20.5; 32.8; 120.0

m – multiplet, sl- singlet large, and t- triplet

Table S3. Chemical shift data (δ ppm) of the d-limonene in $CDCl_3$, 14.1 T at 25 ºC.

m – multiplet. s- singlet

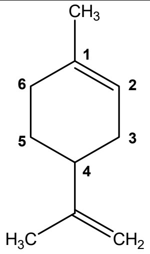

N°	ACD (^{1}H/^{13}C)	δ ^{1}H Multiplicity	δ ^{13}C	HMBC
1	---/**133.23**	-----	**133.15**	----
2	5.30/**120.70**	5.41-5.37 m	**120.8**	120.8; 41.2; 30.6; 30.8; 23.75
3a	1.96-1.92/**30.65**	1.93 m	**30.60**	23.75; 28.0; 41.0; 120.8; 133.1
3b	2.08-2.11/**30.65**	2.05 m	**30.60**	23.75; 28.0; 41.0; 120.8; 133.1
4	2.17/**41.10**	2.09	**41.20**	28.0; 108.5; 120.8; 149.6
5a	1.51/**27.95**	1.51-1.43 m	**28.0**	28.0; 41.0; 133.1; 149.6
5b	--------	1.81-1.76 m	**28.0**	28.0; 41.0; 133.1; 149.6
6a	1.91-1.94/**30.77**	1.93 m	**30.85**	23.75; 28.0; 41.0; 120.8; 133.1
6b	1.99-2.02/**30.77**	2.05	**30.85**	23.75; 28.0; 41.0; 120.8; 133.1
CH_3C1	1.66/**26.63**	1.63 s	**23.75**	23.75; 38.5; 120.8; 1331
CH_{2a}	4.56/**108.80**	4.69 s	**108.5**	108.5; 149.6; 41.2; 20.6
CH_{2b}	4.63/**108.80**	4.69 s	**108.5**	108.5; 149.6; 41.2; 20.6
C=	--- / **149.65**	-----	**149.65**	-----
CH_3	1.69 / **20.67**	1.71 s	**20.6**	20.6; 41.2; 108.5; 149.65

Table S4. Chemical shift (δ ppm) of the hesperidin in DMSO-d6, 14.1 T at 25 ºC.

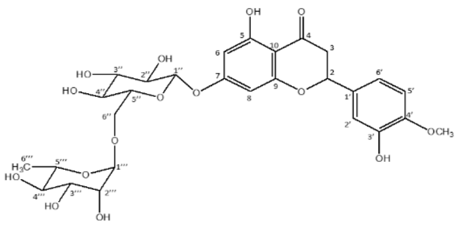

N°	δ ^{1}H Multiplicity (coupling constant in Hz)	δ ^{13}C	HMBC
2	5.5 dd (12.30; 3.26)	78.3	78.3; 42.0; 114.1; 117.9; 196.9
3	3.27 dd (17.19; 12.30)	42.0	42.0; 78.3; 103.3; 130.9; 163.0; 196.9
3	2.78 dd (17.19; 3.26)	42.0	42.0; 78.3; 103.3; 130.9; 163.0; 196.9
4	-----	196.9	-----
5	-----	163.0	-----
6	6.14 d (2.24)	96.3	96.3; 103.3; 163.0; 165.1
7	-----	165.1	-----
8	6.12 d (2.24)	95.5	95.5; 103.3; 162.4; 165.1
9	-----	162.4	-----
10	-----	103.3	-----
1’	-----	130.9	-----
2’	6.95 d (2.0)	114.1	114.1; 78.3; 130.9; 147.9
3’	-----	146.4	-----
4’	-----	147.9	114.1; 112.0
5’	6.94 d (8.39)	112.0	78.3; 112.0; 147.9; 146.4
6’	6.90 dd (8.39; 2.0)	117.9	117.9; 78.3; 114.3; 130.9
OCH_3	3.78 s	55.7	147.9
1”	4.97	99.4	99.4; 75.5; 72.9; 165.1; 163.0; 162.4
2”			
3”			
4”			
5”			
6”a	3.80 dd (11.34; 1.75)	66.0	66.0; 69.6; 75.5; 100.6
6”b	3.42	66.0	75.5; 100.6; 66.0; 69.6
1”’	4.53	100.6	
2”’	3.64	70.2	66.0; 69.6; 76.3
3”’	3.91	70.6	75.5; 100.6
4”’	3.64 ddd	75.5	17.8; 66.0 68.3; 70.6; 75.5; 76.3
5”’	3.15	69.6	17.8; 100.6; 70.6; 75.1
6”’	1.09 d (6.20)	17.8	17.8; 66.0; 75.5; 69.6

d- doublet, dd- double doublet, ddd – double double doublet e s – singlet
